# Analysis of Visual Field Defects Obtained with Semiautomated Kinetic Perimetry in Patients with Leber Hereditary Optic Neuropathy

**DOI:** 10.1155/2018/5985702

**Published:** 2018-03-21

**Authors:** Katarzyna Nowomiejska, Agnieszka Kiszka, Edyta Koman-Wierdak, Katarzyna Tonska, Ryszard Maciejewski, Anselm G. Jünemann, Robert Rejdak

**Affiliations:** ^1^Department of General Ophthalmology, Medical University of Lublin, Lublin, Poland; ^2^Institute of Genetics and Biotechnology, University of Warsaw, Warsaw, Poland; ^3^Human Anatomy Department, Medical University of Lublin, Lublin, Poland; ^4^Department of Ophthalmology, University Eye Hospital, Rostock, Germany; ^5^Department of Experimental Pharmacology, Medical Research Centre, Polish Academy of Sciences, Warsaw, Poland

## Abstract

**Purpose:**

To analyse visual field (VF) defects obtained using semiautomated kinetic perimetry (SKP) in patients suffering from Leber hereditary optic neuropathy (LHON).

**Methods:**

Twenty-two eyes of eleven consecutive LHON male patients with confirmed mitochondrial 11778G>A DNA mutation were prospectively examined with the V4e stimulus of SKP in both eyes. The mean time after the onset of LHON was one year. The area of obtained isopters was measured in square degrees (deg^2^). Additionally, static automated perimetry (SAP) within 30° was performed.

**Results:**

Visual acuity ranged from counting fingers to 50 cm to 0.4. VFs obtained with SKP showed central scotomas in 18 eyes (82%); the periphery of the VF in these eyes remained intact. The mean area of central scotoma was 408.8 deg^2^, and the mean area of the peripheral VF was 12291.1 deg^2^; SAP also revealed central scotoma in these patients. In four eyes (18%) with the worst visual acuity, only the residual central island of VF was found using SKP (mean area 898.4 deg^2^). SAP was difficult to obtain in these patients.

**Conclusions:**

SKP provides additional clinical information in regard to the visual function of LHON patients. SKP enables the quantification of the area of central scotoma, preserved peripheral VF, and residual central island of vision. Using V4 stimulus is especially useful in LHON patients with poor visual acuity, when SAP is difficult to obtain.

## 1. Introduction

Leber hereditary optic neuropathy (LHON) is the most common inherited optic neuropathy [1] with the prevalence ranging from 1 : 31000 to 1 : 50000 [2]. The combination of mitochondrial DNA point mutation (the three most common: 3460G>A, 11778G>A, or 14484T>C) and triggering environmental factors results in disturbed mitochondrial function [3, 4]. Disease onset among LHON carriers is characterized by acute or subacute painless loss of central vision, which is unilateral at the beginning; the fellow eye is usually affected within six to eight weeks [2]. Visual loss is usually severe, dropping to levels of 0.1 or worse in both eyes, making the patients legally blind [5].

Patients with LHON have typical and consistent clinical findings that include loss of visual acuity, dyschromatopsia, decreased contrast sensitivity, and visual field (VF) defects [6]. VF changes are typically bilateral with dense central or centrocecal scotoma with no relationship to the vertical midline and preservation of the peripheral VF. Central scotoma (5°–20° surrounding the point of fixation) usually indicates selective damage of the papillomacular bundle [7].

The VF testing is essential for making a diagnosis and monitoring the visual function of all optic neuropathies, including LHON. Moreover, VF defects can be the only clinical manifestation of LHON, with no fundoscopic changes. However, VF characteristics of LHON patients have not been widely investigated. Static automated perimetry (SAP) [8–12] or Goldmann manual kinetic perimetry [8, 13, 14] has been used so far in the assessment of VF in LHON patients. As the visual acuity in LHON patients is very poor and the VF loss may be severe, Goldmann kinetic perimetry seems to be more suitable, than SAP, for VF testing. Goldmann manual perimetry is easier for the patient; examination may be shortened depending on the alertness of the patient. However, manual kinetic testing has many disadvantages and is examiner-dependent; there is no electronic storage of the results.

Currently, semiautomated kinetic perimetry (SKP) is an alternative for Goldmann manual kinetic perimetry as a more standardized method. The results are digitized, the movement of the stimulus is constant, and the area of the scotoma may be measured in square degrees (deg^2^).

The aim of this study was to evaluate VF results obtained with SKP and V4e stimulus in Polish cohort of patients with diagnosed LHON who carried 11778G>A mutation.

## 2. Material and Methods

We prospectively enrolled eleven consecutive patients (10 males, 1 female; mean age at onset 27 years; range 16–58 years) diagnosed with LHON referred to the Department of General Ophthalmology in Lublin, Poland, between 2011 and 2016. Informed consent was obtained from each individual after an explanation of the nature of the study. The study was approved by the independent ethics committee at the Medical University of Lublin and performed in accordance with the ethical standards laid down in the 2008 Declaration of Helsinki.

The diagnosis of LHON was molecularly confirmed with mtDNA pathogenic mutation (11778G>A in all cases) made in the Institute of Genetics and Biotechnology of the University of Warsaw in Poland. Total DNA of patients was extracted from the peripheral blood according to the standard procedures. The screening for m.3460G>A, m.11778G>A, and m.14484T>C mutations was performed with the methodology described by Tonska and co-authors [15]. VF examinations were performed one year after making the diagnosis of LHON (range 6 months).

All patients had an extensive ophthalmologic examination, including best-corrected visual acuity measurement (decimal Snellen chart), slit-lamp biomicroscopy, intraocular pressure measurement, indirect ophthalmoscopy, and optic nerve head photography. Peripapillary RNFL thickness was measured by an optic disc cube 200 × 200 scan of the spectral-domain ocular coherence tomography (SD-OCT) (Cirrus OCT, 3.0; Carl Zeiss Meditec). Laboratory examinations were conducted to exclude inflammatory optic neuropathy (antibodies against boreliosis and toxoplasmosis). Additionally, visual evoked potential (VEP) examination was performed to assess the neural conduction along the visual pathways and to confirm optic nerve dysfunction. Neurological assessment was performed in each patient, but no neurological dysfunction was found. Magnetic resonance imaging (MRI) examination of the central nervous system was performed to exclude demyelinisation or compressive bilateral optic neuropathy.

### 2.1. Visual Field Examination

Kinetic perimetry of both eyes was performed using an Octopus 900 perimeter (Haag-Streit Inc., Koeniz, Switzerland). Examinations were performed in the mean time period of one year after the onset of LHON. Due to poor visual acuities, the largest and brightest Goldmann perimetry stimulus V4e (area 64 mm^2^, diameter 9.03 mm, brightness < 0 dB, 10.000 asb) was used to assess the peripheral and central VF in LHON patients. The stimulus angular velocity was settled at the level of 5 degrees per second. Both eyes were tested in all patients with the right eye tested first. The fellow eye was patched with an occluder. During VF examination, no optical correction was used. The patient was instructed to look at the fixation point and push the button as soon as he/she could see the stimulus. Stimuli were moved from nonseeing areas towards seeing areas almost perpendicularly towards the presumed scotoma border. Vectors were placed along 24 meridians from the periphery to the center of the VF and additionally from the center to the periphery do delineate central scotoma. A rest break was given when needed. Examination time was measured in minutes. The area of isopters was measured in deg^2^.

Additionally, SAP examination was performed using a Humphrey Field Analyzer (Carl Zeiss, Dublin, Ca, USA) and 30-2 program. The mean deviation (MD) and pattern standard deviation (PSD) parameters were recorded. The MD parameter shows the general light sensitivity of VF; the PSD parameter reflects the local VF defects.

The comparison between two methods (SKP and SAP) was done descriptively by the assessment of VFs by two experienced experts (Katarzyna Nowomiejska and Robert Rejdak). Central scotoma was defined as an isolated scotoma in the circular area between 0° and 10°. The residual central island of vision was defined as an isopter constricted less than the central 30°.

## 3. Results

Patients' visual acuity ranged from counting fingers to 50 cm to 0.4; only the youngest patient (number 1), 16 years old, had the visual acuity better than 0.1 (Table 1). The visual acuity was as follows: 3 eyes with visual acuity counting fingers to 50 cm, 5 eyes with visual acuity counting fingers to 1 m, 7 eyes with visual acuity counting fingers to 2 m, 1 eye with visual acuity counting fingers to 4 m, 5 eyes with visual acuity 0.1, and 1 eye with visual acuity 0.4.

**Figure 1:**
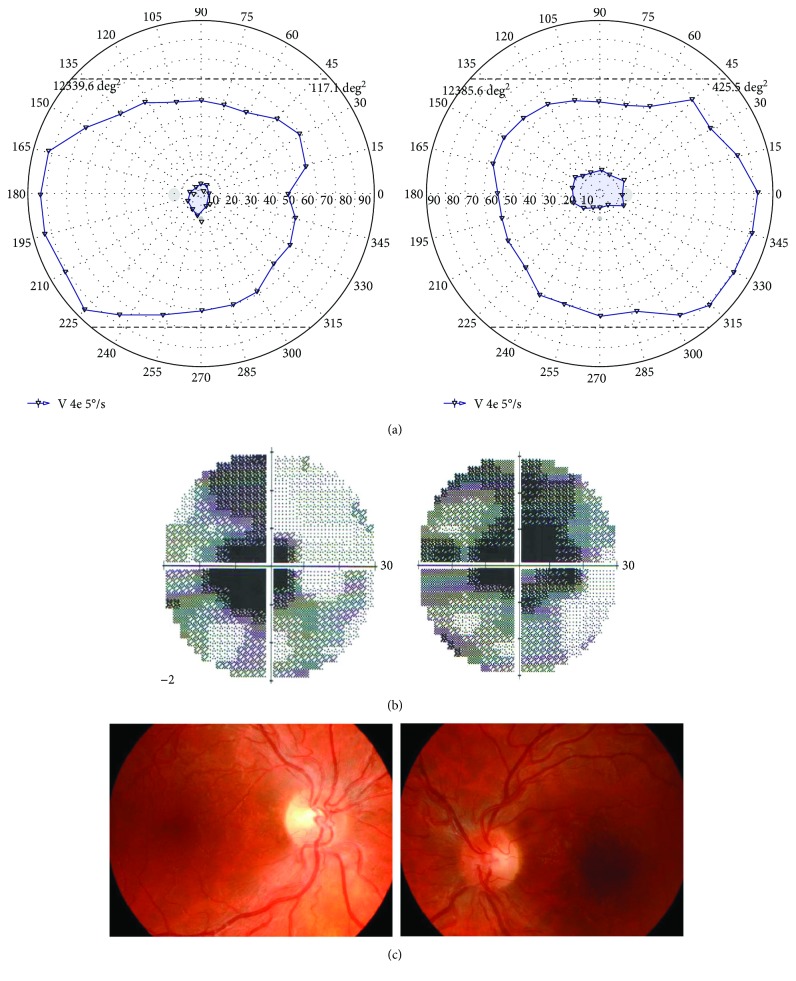
Illustrative case of LHON patient with central scotoma. (a) VF results of both eyes (right eye on the right and left eye on the left) obtained with semiautomated kinetic perimetry and V4e stimulus moved with the speed of 5 degrees per second in patient with LHON. Visual acuity was counting fingers to 2 meters in both eyes. Central scotoma and peripheral residual VF measured in square degrees (deg^2^). (b) VF results of both eyes (right eye on the right and left eye on the left) obtained with static automated perimetry in patient number with LHON. (c) Fundus pictures (right eye on the left and left eye on the right) of patient number 8 showing swelling of the retinal nerve fibers involving the superior and inferior arcades around the optic disk, as well as retinal vessel tortuosity.

**Figure 2:**
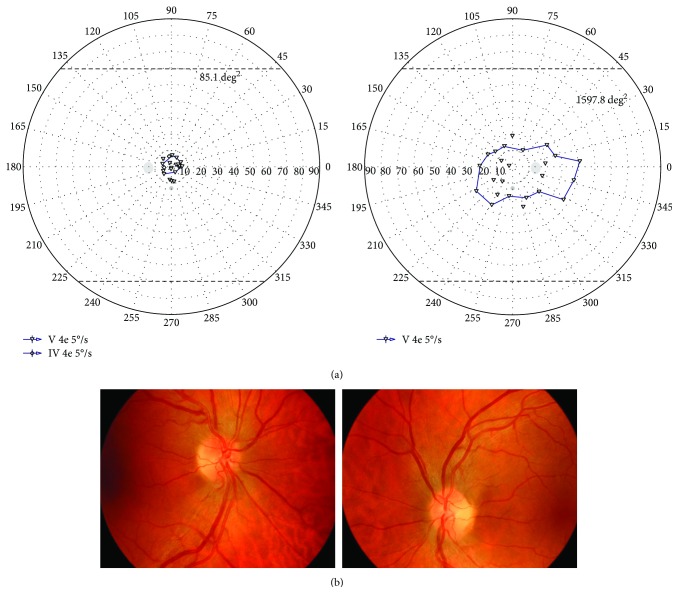
Illustrative case of LHON patient with residual central island of vision. (a) Visual field result of both eyes (right eye on the right and left eye on the left) obtained with semiautomated kinetic perimetry and V4e stimulus moved with the speed of 5 degrees per second in patient with LHON. Visual acuity was counting fingers to one meter in both eyes. Central residual visual field measured in square degrees (deg^2^). Static automated perimetry was not obtained due to poor visual acuity. (b) Fundus pictures (right eye on the left and left eye on the right) of patient number 10 showing swelling of the retinal nerve fibers involving the superior and inferior arcades around the optic disk.

VF results obtained with V4e stimulus included 14 central scotomas (7 patients) (82%) (Figure 1) and the residual central island of vision (Figure 2) in 2 patients (4 eyes) (12%) with the worst visual acuity (number 3) counting fingers to 30 cm. All VF patterns were present binocularly and were identical in both eyes of each patient. The mean area of central scotoma was 408.8 deg^2^ (range 11.8–621.4 deg^2^), and the mean area of the peripheral VF of 18 eyes was 12291.1 deg^2^ (range 11290.7–14020.2 deg^2^). The MD in SAP in this group was −16.24 dB, and the mean PSD was 8.89 dB. There were central scotomas found in SAP by 2 experts.

The mean area of the central VF island of remaining 4 eyes with the worst visual acuity was 898.4 deg^2^. SAP was not performed in these patients because of poor visual acuity.

Most of the patients (10) had no family history of LHON. The onset of the VF loss occurred first monocularly and then extended to the second eye. There were abnormalities in the fundoscopy in 5 patients, hyperemia of the optic disc, tortuosity of vessels, and swelling of the retinal nerve fiber layer (RNFL) around the optic disc. Mean intraocular pressure was 17 mmHg (range 11–19 mmHg). The mean peripapillary RNFL layer was 108.9 *μ*m (range).

VEP showed delayed P100 latencies for large check sizes and no recordable responses for small check sizes in all patients.

## 4. Discussion

The present study demonstrates the usefulness of SKP in patients evolving extensive bilateral VF defects due to LHON. To our knowledge, this is the first study describing VF defects using SKP and SAP in LHON patients.

Our Polish LHON cohort is comparable with other published case series, with a predominance of the 11778G>A mutation, most patients becoming affected in their twenties, and males having a higher risk of visual loss than female carriers [5]. Among three the most common mutations responsible for LHON, mutation 11778G>A carries the worst visual prognosis with the visual recovery rate of 4–7% [16] and large absolute central scotoma [17]. In our series of eight patients examined with the V4e stimulus of SKP, one suffered from constriction of the VF and the residual central VF island.

The anatomical basis of centrocecal sotoma has to be established [18]. Centroceal scotoma extends from the fixation point towards the blind spot and is due to the involvement of the papillomacular bundle arising from the fovea towards the optic disc [19]. The “papillomacular bundle” of optic nerve fibers was originally described in autopsy studies of toxic amblyopia and not normal anatomy [18].

In LHON, retinal ganglion cells (RGC) seem to be selectively vulnerable to mitochondrial dysfunction [20, 21]. It is also responsible for the preservation of pupils' reaction in LHON patients. The papillomacular bundle is affected first, and its apoptosis and axonal swelling are responsible for the central VF loss, as well as dyschromatopsia [20]. This process is due to the small diameter of nerve fibers (parvocellular population) and dependence on the mitochondria [20]. Small diameter fibers are very energy dependent for maintaining efficient axoplasmic transport. The concentration of the mitochondria is higher in unmyelinated RGC than in myelinated fibers behind the lamina cribrosa. Long-standing damage of RGCs extends to the rest of the nerve, leading to optic atrophy [20].

Most of the patients with LHON reported in the literature had central or centrocecal scotoma, [6, 22]; however, VF defects mimicking bitemporal hemianopsia have also been described in LHON patients [23]. Japanese authors found that the central-most temporal VF obtained with SAP 30° appears to be the most susceptible to damage in the eyes of patients who experienced monocular loss of vision in the fellow eye [11]. Ran and coworkers [24] investigated VFs in LHON within a cohort of 32 patients (49 eyes) within 6 months of the follow-up. VF defects revealed central scotoma in 26 eyes (53.1%), paracentral scotoma in 12 eyes (24.5%), cecocentral defects in 6 eyes (12.2%), blind spot enlargement in 3 eyes (6.1%), and quadrantanopia in 2 eyes (4.1%) within 1 week after onset. After 6 months, central isopter constriction was observed in 18 eyes (36.7%), diffuse defects in 21 eyes (42.9%), cecocentral defects in 3 eyes (6.1%), hemianopia or quadrantanopia in 5 eyes (10.2%), and central scotoma in 2 eyes (4.1%). They observed that LHON at different stages was characterized by different focal VF defects: VF defects in LHON patients within 1 week after onset were mostly central or paracentral scotoma, which was enlarged around the cecocentral defect or connected to form a blind spot after 3–6 months. In the present study, we have analysed VF defects after a mean period of one year after the onset of LHON.

In our study, we have performed both kinetic and static perimetries in a group of patients suffering from LHON. However, there are many disparities between static and kinetic perimetries.

Kinetic perimetry has an extent of 90° of the visual field including the far periphery, as it enables to cover large areas of the visual field in a fairly short time. SAP is not applicable for determining the entire 90° extent of VF because of the large number of test locations and the variability of peripheral differential luminance threshold. Thus, it produces extended examination time and fatigue [25].

There are different indications for certain types of perimetry. Static perimetry within 30° is a standard in glaucoma diagnostics and monitoring; kinetic perimetry is preferred in neuroophthalmological conditions, extended visual field defects, and low visual acuity. If there is a suspicion of functional visual loss, kinetic perimetry is the examination of choice [26].

As kinetic and static perimetries are based on completely different principles, it is very difficult to compare them quantitatively. Thus, only qualitative description is possible. In the present study, VFs obtained using SKP and SAP were assessed by two experienced experts. It was performed in the same manner as in the study comparing static and kinetic perimetries [27]. We observed extensive central scotoma obtained with both methods in nine of eleven LHON patients. Moreover, in the remaining two patients, static perimetry was impossible to obtain due to very poor visual acuity. Some authors reported that it is not possible to perform SAP reliably in LHON patients due to poor visual acuity [28]. Deficiencies in high spatial frequency contrast sensitivity have also been reported in LHON patients [29].

Kinetic perimetry seems to be better suited for low vision patients, as there are moving stimuli of large size. If there are large, steeply borded scotoma, moving stimuli make possible edge detection and pattern definition. Kinetic targets are closer to reality than static ones; moreover, the examination may be adjusted to the patient's alertness.

Goldmann manual kinetic perimetry is very rarely used nowadays, as it is manually operated and is charged with examiner bias. SKP offers the possibility to measure the area of scotoma, including central scotoma in square degrees. In the present study, we used the largest Goldmann stimulus V4e; it is usually used in patients with poor visual acuities. The area of isopters obtained by SKP has already been measured in patients suffering from advanced glaucoma, retinal dystrophies, and neuroophtalmological diseases [30], as well as epilepsy patients treated with vigabatrin [31]. The results of SKP have been already compared with Goldmann manual perimetry in 77 patients with different neuroophtalmic conditions by the author of the above manuscript. In order to compare the location and size of the corresponding isopters obtained with both methods, intersection areas of superimposed isopters were expressed as the percentage of union areas. Isopters obtained with Goldmann perimetry were generally smaller by 20% [31].

The best visual acuity (0.4) was found in the youngest patients—16 years old. It has been already described that younger age is a good predictive factor for the visual acuity [32]. The average onset of LHON is at 24.3 years of age among males and at 31.8 years among females. In our study, there was one man above this aged 58.

Both VF and visual acuities are very important indicators in regard to everyday life activities of LHON patients. Preservation of the peripheral VF makes affected individuals able to walk around independently.

Currently, no effective treatment for LHON has been developed; however, gene therapy is under investigation in hereditary eye diseases [33]. SKP may be a useful diagnostic method of visual function assessment to monitor the effectiveness of treatment of novel methods. So far, only SAP was used in these patients [34].

We conclude that SKP adds additional information in regard to visual function in LHON patients. Using V4e stimulus enables the assessment of the preserved peripheral VF, even if central visual acuity is very poor. We believe that SKP may be a useful tool in making a diagnosis, follow-up examinations, and potential future treatment methods. However, LHON is a rare disease; thus, the present study has been limited by the small number of cases.

## Figures and Tables

**Table 1:** Gender, age, visual field defect obtained using semi-automated kinetic perimetry (SKP), and visual acuity of patients with diagnosed Leber hereditary optic neuropathyCF-counting fingers).

Number	Age at onset	Visual field defect of the right eye	Visual field defect of the left eye	Visual acuity right eye (Snellen)	Visual acuity left eye (Snellen)
1.	16	Central scotoma	Central scotoma	0.1	0.4
2.	25	Central scotoma	Central scotoma	CF to 1 m	0.1
3.	26	Residual island	Residual island	CF to 0.3 m	CF to 0.3 m
4.	22	Central scotoma	Central scotoma	CF to 1 m	CF to 1 m
5.	26	Central scotoma	Central scotoma	CF to 1 m	CF to 4 m
6.	20	Central scotoma	Central scotoma	CF to 1 m	0.1
7.	28	Central scotoma	Central scotoma	CF to 1 m	CF to 1 m
8.	19	Central scotoma	Central scotoma	CF to 2 m	CF to 2 m
9.	22	Central scotoma	Central scotoma	CF to 2 m	CF to 2 m
10	58	Residual island	Residual island	CF to 1 m	CF to 1 m
11	29	Central scotoma	Central scotoma	CF to 1 m	0.1
